# Objective Longitudinal Monitoring of Burn Wound Area Using 3D Surface Scanning: A Pilot Study

**DOI:** 10.3390/ebj7010015

**Published:** 2026-03-06

**Authors:** Bibiána Ondrejová, Katarína Dudová, Monika Michalíková, Lucia Bednarčíková, Jozef Živčák, Tomáš Demčák, Peter Lengyel

**Affiliations:** 1 Department of Biomedical Engineering and Measurement, Faculty of Mechanical Engineering, Technical University of Košice, Letná 1/9, 042 00 Košice, Slovakia; katarina.dudova@tuke.sk (K.D.); monika.michalikova@tuke.sk (M.M.); lucia.bednarcikova@tuke.sk (L.B.); jozef.zivcak@tuke.sk (J.Ž.); 2 Burns and Reconstructive Plastic Surgery Clinic, AGEL Hospital Košice Šaca, Lúčna 512, 040 15 Šaca, Slovakia; tomas.demcak@nke.agel.sk (T.D.); peter.lengyel@nke.agel.sk (P.L.)

**Keywords:** burn, 3D scanning, wound analysis

## Abstract

Background: Burn assessment traditionally relies on visual inspection and 2D estimation, which introduces substantial variability in determining wound size and healing progression. Three-dimensional (3D) surface scanning offers a more objective alternative, yet the clinical utility of area-based metrics obtained from 3D surface data remains insufficiently defined. This pilot study aimed to evaluate structured-light 3D scanning for objective longitudinal quantification of the burn wound surface area and a description of area-based healing dynamics derived from repeated measurements. Methods: Eighteen patients with 43 acute thermal burns underwent serial structured-light scanning, followed by manual segmentation of wound regions and the calculation of absolute and percentage area reduction as well as TBSA-normalized metrics. Longitudinal monitoring was performed by comparing sequential 3D surface models acquired at defined clinical follow-ups, enabling the calculation of absolute area change (ΔA), percentage reduction, daily healing rate, and ΔTBSA%. Results: Baseline wound areas ranged from 7.27 to 2137.98 cm^2^. Percentage area reduction ranged from 5.25% to 92.30%. The overall reduction in burn burden (ΔTBSA) ranged from 0.07% to 12.94%. Large wounds tended to show rapid absolute area reduction (>100–300 cm^2^/day) during early follow-up, while small superficial burns frequently achieved >80% reduction within 10–15 days. Conclusions: These findings suggest that 3D surface scanning may support the objective longitudinal assessment of burn wound healing. This pilot provides a basis for future studies evaluating additional topographic parameters and broader clinical applications.

## 1. Introduction

Burn injuries represent a major global health burden, with an estimated 11 million cases annually, often resulting in extensive tissue damage, functional impairment, and psychological distress. Accurate and objective assessment of burn wound severity and healing progression is critical for guiding clinical decision-making and evaluating treatment efficacy [1].

Traditional assessment methods—such as the Rule of Nines, the Lund–Browder chart, and 2D photography—rely heavily on clinician estimation, leading to inter-observer variability and limited reproducibility, particularly for irregularly shaped or curved anatomical surfaces [2].

In recent years, three-dimensional (3D) imaging technologies have emerged as highly promising tools for burn assessment, enabling the precise quantification of wound geometry, surface area, and volume. Structured-light and stereophotogrammetric 3D scanners, such as the Artec Eva (Artec 3D, Luxembourg, Luxembourg), have demonstrated sub-millimeter precision, non-invasive operation, and rapid acquisition capabilities suitable for use in sterile hospital environments [3]. Compared to manual tracing and 2D imaging, 3D scanning provides higher accuracy in measuring the burn area and contraction dynamics over time [4]. This quantitative advantage makes it a valuable tool for both the acute management and longitudinal follow-up of burn patients. Beyond surface area estimation, 3D imaging facilitates volumetric and topographic analysis, allowing clinicians to visualize and measure wound depth and healing trajectories in unprecedented detail. Studies have confirmed that 3D scanning can accurately track healing progression, even in complex wounds, and support the evaluation of interventions such as skin grafts and bioengineered dressings [5,6]. Its integration into digital wound monitoring systems enhances the accuracy of clinical documentation and may improve interdepartmental communication and treatment standardization [7].

Moreover, 3D-based technologies extend into reconstructive and rehabilitative applications, including the design of patient-specific compression garments, splints, and surgical planning models [8,9]. When combined with 3D printing, these imaging systems support personalized interventions that adapt to the patient’s anatomy and evolving wound morphology [10,11,12].

The aim of this pilot study is to determine which surface-area-based parameters of burn healing can be reliably derived from structured-light 3D scans and how these measurements may complement standard clinical assessment. Rather than evaluating all aspects of 3D scanning feasibility, this study focused specifically on the longitudinal monitoring of wound area derived from repeated 3D surface models.

## 2. Materials and Methods

A total of 18 patients with 43 (average wound per patient ≈ 2.4) acute thermal burns were prospectively enrolled in the study conducted between February 2024 and June 2025. The mean age of the patients was 48.5 ± 17.3 years. All participants presented with acute burns ranging from superficial to full-thickness, affecting one to four anatomical locations per patient. The initial burn extent, expressed as total body surface area (TBSA), averaged 17.3 ± 12.8%. For each patient, the following demographic and clinical variables were recorded: age, sex, height, weight, burn location(s), depth of the lesion, and treatment modality (conservative versus surgical). Burn depth was clinically classified as superficial (I), partial-thickness (II), and full-thickness (III) burns according to clinical assessment by the attending burn surgeon; depth assessment was based solely on clinical evaluation and not on 3D scanning data. Partial-thickness burns included both superficial dermal (IIa) and deep dermal (IIb) injuries. For readability, numerical notation (I–III) is retained in the tables and figures. Patients were consecutively recruited from the burn unit based on the presence of burn wounds with clearly identifiable margins. Once a patient was enrolled, all burn wounds present on the patient were included in the analysis, without wound-level selection.

Ethical approval was obtained from the institutional ethics committee prior to study initiation, and written informed consent was secured from all participating individuals. The study was reviewed and approved by the Ethics Committee of AGEL Hospital Košice-Šaca (Slovakia) under protocol number 17-2023. A structured set of questionnaires was developed for physicians and nursing staff to gather comprehensive clinical information.

The physician questionnaire included items related to patient demographics, burn extent assessed by standard clinical methods, burn depth classification, and the potential need for surgical intervention. This collaboration with the Department of Burns and Reconstructive Surgery at AGEL Hospital Košice-Šaca enabled access to relevant clinical data and provided a robust foundation for scientific investigation. The comprehensive design of the data-collection process, together with close cooperation with clinical professionals, established an essential framework for the subsequent analysis and interpretation of the results.

### 2.1. 3D Scanning Methodology

**Figure 1:**
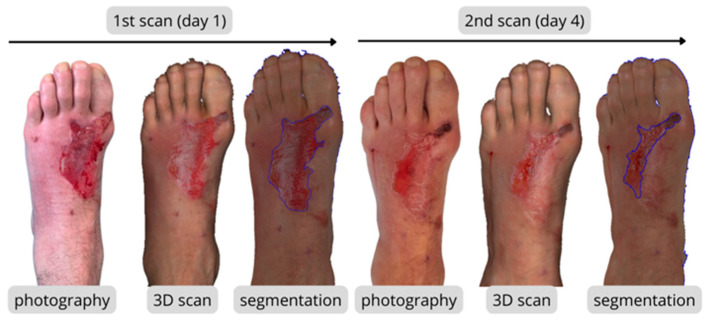
Representative example of 3D surface scanning, clinical photography, and burn wound segmentation performed during longitudinal follow-up.

The 3D scanning methodology was used exclusively for surface geometry acquisition and wound area measurement Burn surfaces were quantified using a structured-light 3D scanner Artec Eva, featuring a 3D resolution of 0.2 mm, accuracy up to 0.1 mm, 16 FPS acquisition rate, and 1.3 MP texture resolution.. Scans were routinely acquired during dressing-change sessions, following dressing removal and standard wound preparation. To minimize artifacts, scanning was performed under diffuse clinical lighting. When necessary, residual ointments or moisture were gently removed to reduce surface glare and prevent distortion of the reconstructed geometry. No major light-related artifacts were observed that would compromise the area calculations. Patients were positioned in a stable and comfortable posture. The scanner was operated at approximately 0.5 to 1 m from the wound surface. Acquisition was restricted to clinically affected regions, with scanning performed only in areas containing burn wounds. When burns involved multiple anatomical locations (e.g., upper and lower limbs), each region was scanned separately. Individual scans were defined according to anatomical boundaries and visible wound extent, so that wound regions were captured as coherent units without arbitrary interruption across anatomical segments. Within each anatomical region, the scanner was moved continuously, and adjacent passes included partial overlap to allow for reliable automatic alignment and merging during post-processing in Artec Studio 13 (Artec 3D, Luxembourg, Luxembourg). Each burn was captured from multiple angles, with a minimum coverage of 180° for planar regions and up to 360° for limbs or anatomically complex areas, depending on the extent and irregularity of the injury. Follow-up scans were integrated into routine clinical care and acquired during dressing changes or upon clinically observable wound progression. Because the study followed a pragmatic clinical design, follow-up intervals were not strictly predefined, leading to minor variability in the timing of sequential scans. Standardized clinical photography (Figure 1) was performed using consistent patient positioning, camera distance, and viewing angle under comparable lighting conditions. These photographs served as visual references to ensure reproducible localization of the scanned wound area during subsequent follow-up sessions. To ensure comparability, each patient was positioned identically during all scanning sessions; for example, if the arm was extended during the initial scan, the same position was reproduced at every subsequent scan. The resulting 3D models provided high-precision geometric data suitable for consistent surface area extraction and temporal comparison.

### 2.2. Segmentation of Wound Area

**Figure 2:**
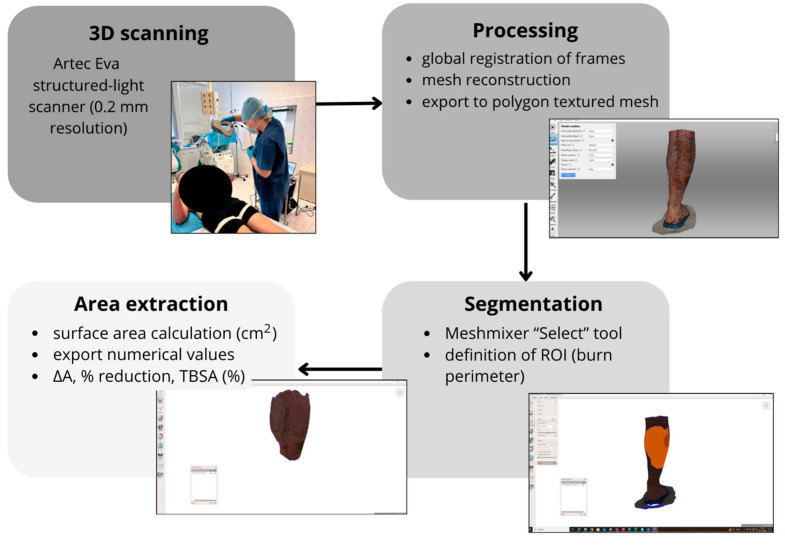
Workflow for quantitative extraction of the burn wound surface area from 3D scans.

Raw 3D scan data were processed in Artec Studio 13, where sequential steps included image registration, mesh reconstruction (performed without watertight filling to preserve the native wound geometry), and artifact removal to eliminate scanning noise and environmental distortions. Following preprocessing, wound segmentation was performed by a single biomedical engineer under the guidance of the attending burn specialist. Segmentation was conducted in Meshmixer 3.5 (Autodesk Inc., San Rafael, CA, USA) using the Select tool to accurately delineate the wound perimeter, guided by the texture information visible on the 3D scan to differentiate viable skin from the burn surface (Figure 2). For each case, a precise region of interest (ROI) representing the burn boundary was manually defined on the reconstructed mesh. From the segmented 3D model, the wound surface area (mm^2^) was quantitatively extracted for the subsequent analysis of healing progression.

### 2.3. Data Analysis

Given the pilot nature of the study and the limited sample size (18 patients), the analysis was restricted to descriptive statistics. Continuous variables were presented as the mean, median, standard deviation, and range, and categorical variables as counts and percentages. No formal hypothesis testing was performed; group comparisons (by burn degree and mechanism of injury) are shown only graphically to illustrate trends.

#### Area-Based Quantitative Metrics

All metrics used for the quantitative analysis of burn healing were derived exclusively from the wound surface area (cm^2^) obtained from the segmented 3D scans. The following area-based metrics are commonly used in quantitative wound-healing analysis [13,14,15].

Wound Area:

(1) $$A_{t},$$

primary surface area measurement of the burn at *t*—time interval from baseline to time *t* (days).

Absolute Area Reduction:

(2) $$\Delta A=A_{0}-A_{t},$$

absolute decrease in *A*_0_—wound area at baseline (cm^2^) and *Aₜ*—wound area at time *t* (cm^2^).

Percentage Area Reduction:

(3) $$Area\;Reduction\;(\%)=\frac{A_{0}-A_{t}}{A_{0}}\times 100,$$

percentage decrease in wound area relative to the initial area.

To express wound size in clinically standardized burn units, the wound surface area (cm^2^) obtained from the 3D models was converted to TBSA-equivalent percentages. The 3D scanning methodology was applied only to local burn wounds and was not used for whole-body TBSA assessment required for acute clinical decision-making.

For each patient, the total body surface area (BSA, m^2^) was calculated individually from height and weight using the Du Bois formula [16]. TBSA (%) and ΔTBSA (%) were then computed as the ratio of the total wound area (or its reduction over time) obtained from the 3D models to the individual BSA.

The individual body surface area (BSA, m^2^) was calculated using the Du Bois formula:

(4) $$BSA\;(m^{2})=0.007184\times W^{0.425}\times H^{0.725}$$

The resulting BSA (m^2^) was converted to cm^2^ and subsequently used to compute the TBSA% from the wound area values. The overall reduction in TBSA% between the first and last scan was reported as:

(5) $$\Delta TBSA\;\left(\%\right)=\frac{A_{0}-\;A_{last}}{BSA}\;\times 100,$$

This metric allowed for the normalization of wound size relative to individual body surface area and provided a clinically interpretable indicator of healing progression.

Early-phase healing was evaluated using the first available follow-up scans obtained during the initial post-injury period (typically within approximately 3–10 days, depending on clinical availability). This interval was selected to capture early post-injury healing dynamics; however, depending on burn depth, some superficial dermal burns may have already shown advanced epithelialization at later time points within this range. For each wound, early-phase healing was quantified using two complementary metrics derived from 3D surface area changes:

(6) $$Early-phase\;Reduction\;(\%)=\frac{A_{0}-A_{t}}{A_{0}}\times 100,$$

where t represents the time of the first follow-up scan, performed during the early post-injury period according to clinical availability.

Daily healing rate enabled the normalization of early healing dynamics for wounds captured at different time intervals:

(7) $$Daily\;Healing\;Rate\;({cm}^{2}/day)=\frac{A_{0}-A_{t}}{t}\times 100$$

## 3. Results

### 3.1. Patient Characteristics

A total of 18 patients (4 females, 14 males) with acute burns were included in this pilot study. The mean age was 48.5 ± 17.3 years (range 19–79). Scald injuries were the most common (*n* = 7; 38.9%), followed by explosion-related burns (*n* = 6; 33.3%) and fire-related burns (*n* = 5; 27.8%). Burn depth varied across the cohort, with partial-thickness burns (including both superficial (IIa) and deep dermal (IIb) injuries) representing the majority of cases, while mixed-depth injuries (I–III, II–III) occurred in 6 patients. Surgical intervention (excision + grafting) was required in 5 patients (27.8%). The initial total body surface area (TBSA) estimated from the 3D-wound area ranged from 0.2% to 30.5%, with a median of approximately 3.6%. Detailed demographic and clinical data are shown in Table 1.

### 3.2. Wound-Level Characteristics from 3D Area Measurements

Across all patients (*n* = 18) and all quantified burn wounds (*n* = 43), substantial variability was observed (Table 2) in the baseline wound size, healing dynamics, and the corresponding reduction in burn burden expressed as ΔTBSA%. Baseline wound areas (A_0_) ranged from 7.3 cm^2^ to 2137.9 cm^2^, reflecting the heterogeneity of burn extent across the cohort.

The absolute reduction in wound area (ΔA) varied widely between wounds, from minimal changes (e.g., 1.8 cm^2^ in P04–LLL) to extensive decreases exceeding 900 cm^2^ (e.g., P14–LUL: 865.9 cm^2^, P17–RLL: 854.9 cm^2^, P09–RLL: 934.0 cm^2^, P12–RLL: 1099.9 cm^2^). When normalized to the baseline size, the percent area reduction ranged from only 5.3% (P11–Torso) to over 90% (P13–Torso: 92.3%), with the majority of wounds falling between 25 and 75% reduction. Notably, wounds that underwent surgical intervention (e.g., P01, P10, P12, P14, and P16) tended to show higher reduction percentages, which may reflect surgical closure and graft incorporation, leading to a marked decrease or complete disappearance of measurable open wound area in some cases.

At the patient level, the total reduction in burned body surface area (ΔTBSA%) provided an integrated measure of healing across all wounds. ΔTBSA% ranged from 0.07% to 12.9%, with the highest values observed in patients with initially extensive or multi-site injuries (P09: 12.9%, P17: 9.2%, P12: 7.0%, P14: 6.4%).

These patients presented with multiple large wounds and showed substantial overall decrease in burn burden during the follow-up period.

In contrast, patients with small and localized burns (e.g., P15: 0.07%, P16: 0.12%, P08: 0.25%) demonstrated correspondingly low ΔTBSA%, despite a sometimes high relative area reduction at the individual-wound level. This highlights that ΔTBSA% is primarily a measure of global burn burden, complementary to local wound-specific metrics such as percent reduction or absolute area change.

Overall, the chart exhibited substantial variability in healing rates:

- Median percent reduction across all wounds over the individual follow-up periods (from baseline to the last available scan) was approximately 57%.
- Large wounds (>1000 cm^2^) demonstrated rapid wound-area reduction within the early follow-up period (approximately 7–14 days).
- Small superficial burns showed the highest percent reduction (>80–90%) within the early follow-up period (approximately 7–14 days).

The wide range of healing dynamics emphasizes the benefit of objective area-based monitoring.

### 3.3. Differences by Burn Degree and Mechanism of Burn

**Figure 3:**
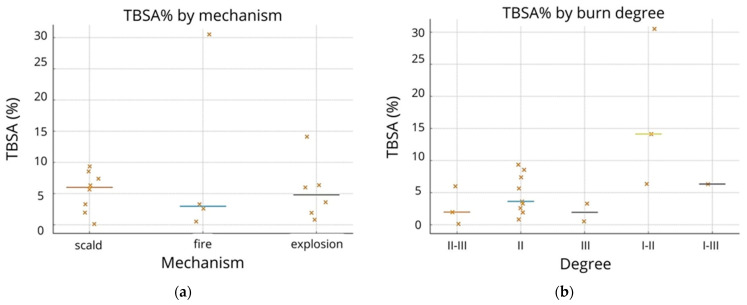
Swarm plot illustrates the distribution of TBSA (%) across the (**a**) burn mechanism and (**b**) burn degree.

Figure 3a shows the distribution of TBSA (%) across scald, fire, and explosion injuries. Scald burns presented the narrowest range (≈1–9%), typically involving localized regions. Fire-related burns displayed the greatest variability, including the most extensive case in the cohort (30.5%). Explosion injuries demonstrated mixed patterns, generally clustering at 1–6%, with one outlier (>14%).

Median TBSA across mechanisms remained comparable (≈3–6%), indicating that mechanism alone does not adequately predict burn extent.

Figure 3b compares the TBSA values across four burn-depth groups. TBSA did not cluster clearly across depth categories. Several of the largest TBSA injuries (>10%) were superficial and partial-thickness burns (I, IIa, IIb). Full-thickness (III) injuries in this cohort were relatively small in TBSA (<4%).

Percent reduction showed wide inter-patient variability within each mechanism and burn-depth subgroup. 

Explosion-related burns tended to heal more uniformly (generally 25–70%), scald injuries frequently reached a >70% reduction, whereas fire-related burns demonstrated highly variable wound-area reduction patterns. Depth-related differences were subtle, with no clear separation between the II and II–III groups. Superficial and partial-thickness burns (I, IIa, IIb) showed some of the largest percent reductions, consistent with higher epithelialization capacity.

### 3.4. 3D Healing Trajectories

**Figure 4:**
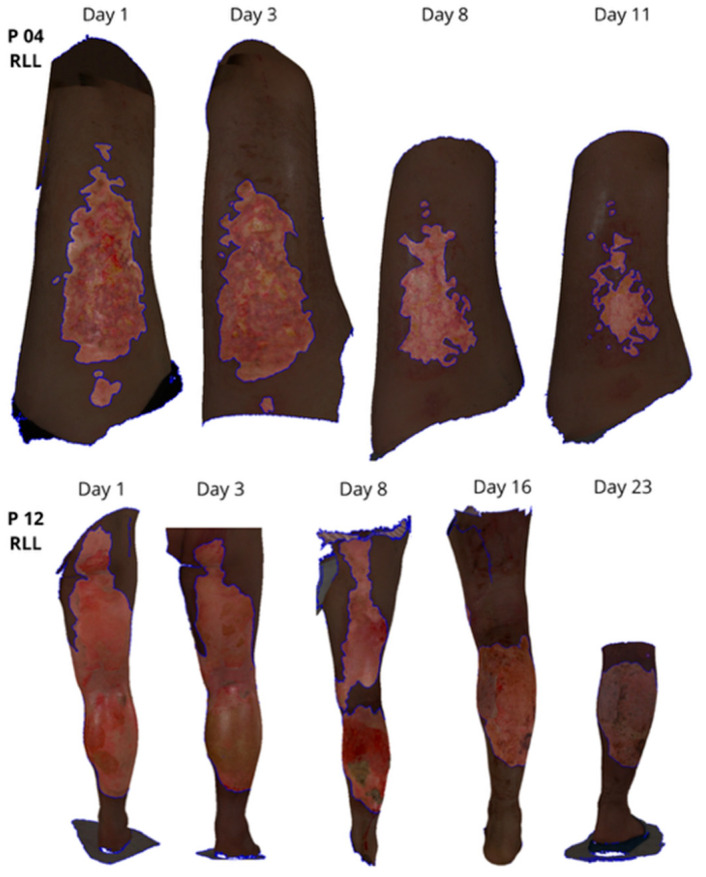
Sequential 3D surface scan wound models of patients 4 and 12 demonstrating burn healing over time.

Sequential 3D scans allowed for detailed monitoring of wound area progression (Figure 4). Several patients (e.g., P12, P17) exhibited non-linear healing dynamics with rapid initial area decline followed by a slower plateau.

In contrast, small-area burns (e.g., P15, P18) demonstrated early near-complete re-epithelialization. These trajectory patterns underline the clinical value of repeated 3D documentation of representative 3D models (Figure 4), illustrating how progressive reduction in wound area and changes in wound margins can be visualized and quantified over consecutive scans.

### 3.5. Early-Phase Healing Dynamics

Across all wounds, early-phase healing exhibited substantial variability. Several general patterns were observable:

#### 3.5.1. Rapid Early Area Reduction in Small Partial-Thickness Burns

Superficial and partial-thickness burns (I, IIa, and IIb) frequently achieved a 30–60% reduction in wound surface area within the first 3–7 days.

Daily healing rates in these wounds commonly ranged from 5 to 20 cm^2^/day, with some very small wounds (e.g., P04–LLL, P15–LLL) nearing complete epithelialization within the early interval.

#### 3.5.2. Large Wounds Showed High Absolute but Moderate Relative Reduction

Extensive burns (>500–1000 cm^2^), such as those of P09, P12, and P17, demonstrated rapid early reduction in absolute terms (≥100–300 cm^2^/day) but showed more moderate relative reduction (15–35% over 5–10 days).

#### 3.5.3. Depth-Dependent Differences Emerged Early

Full-thickness or mixed-depth wounds (IIb–III and III) tended to show slower early relative reduction, typically <20–40% within the same timeframe. Daily area-reduction rates were also lower relative to the initial size, indicating limited early epithelial migration and slower wound-bed remodeling.

#### 3.5.4. Surgically Treated Wounds Showed Pronounced Early Reduction

Wounds that underwent surgical excision and grafting (e.g., P01 RLL, P10 torso, P12 RLL) demonstrated the highest early reductions (>70–85%), which corresponded to the removal of necrotic tissue and early graft take rather than intrinsic spontaneous healing. This confirms that 3D scanning captures the immediate morphological effects of surgical management.

## 4. Discussion

This pilot study demonstrates that 3D surface scanning provides a feasible and objective method for the quantitative monitoring of burn wound healing. By analyzing 43 wounds in 18 patients, we showed that serial 3D models enable the accurate measurement of wound area, percent reduction, and burn surface involvement (TBSA%), offering clinically relevant metrics that complement traditional visual assessment.

The variability observed in wound size, area-reduction dynamics, and TBSA across different burn mechanisms and depths highlights the limitations of subjective estimation and underscores the value of quantitative digital documentation.

A major finding of this study is the substantial heterogeneity in healing behavior, even among wounds of similar burn depth or mechanism. Percent reduction ranged from 5% to over 90%, and absolute reductions exceeded 1000 cm^2^ in the largest injuries. These differences reflect the complex interaction between burn depth, wound geometry, patient factors, and treatment strategy. Notably, superficial and partial-thickness burns (I, IIa, IIb) were often associated with the highest relative reductions, consistent with the higher epithelialization potential of these wounds. Conversely, full-thickness or mixed-depth burns showed more variable and slower area-reduction patterns, in line with the published evidence indicating that deeper burns rely primarily on surgical closure or delayed tissue remodeling rather than rapid epithelial migration.

Another key observation is that within this pilot cohort, burn extent (TBSA%) did not correlate with the clinically assessed burn degree (depth classification). Several of the most extensive injuries in this cohort were superficial or partial-thickness burns, while full-thickness burns tended to be more localized. This observation is consistent with previously reported clinical experience suggesting that TBSA and burn depth may represent distinct aspects of burn severity; however, this interpretation should be limited to the present pilot cohort. The use of 3D scanning allowed for the quantitative estimation of TBSA independent of clinical visual estimation, which may be useful given the reported variability of traditional methods such as the Rule of Nines or Lund–Browder chart [17,18].

Comparison of TBSA across burn mechanisms demonstrated variability within this cohort. Fire-related burns showed the widest range of surface involvement including the largest individual case (>30% TBSA). Scald burns tended to present with smaller affected areas, while explosion burns demonstrated heterogeneous patterns. These trends should be interpreted as descriptive observations within the studied cohort rather than generalizable conclusions, but they may help inform hypotheses for future larger studies.

The feasibility of using the Artec Eva scanner in a clinical environment was another important outcome. Scanning before dressing changes did not prolong the clinical workflow and was well-tolerated by all patients. The ability to repeatedly capture surface geometry with high spatial resolution (0.1–0.2 mm) enabled the reliable segmentation of wound boundaries based on texture cues. This supports the growing body of evidence that structured-light 3D scanning provides an accurate and reproducible alternative to traditional planimetric or photographic measurement methods.

Furthermore, the method provides actionable information for individualized treatment planning. Quantitative monitoring of wound area over time may help clinicians identify atypical healing trajectories, assess the effectiveness of dressings or grafts, and support early intervention in cases of delayed healing. In future applications, this methodology could be used to evaluate advanced interventions, such as personalized 3D-printed burn masks or customized pressure garments, by providing objective before–after comparisons within the same anatomical region.

Recent evidence suggests that cutaneous functional units (CFUs) may represent a more sensitive indicator of post-burn functional impairment than global TBSA alone, particularly in relation to joint mobility and contracture risk. Although CFU-based analysis was beyond the scope of this pilot study, structured-light 3D surface scanning provides a highly suitable technological platform for the future integration of functional-unit-based assessment. Unlike conventional 2D documentation, 3D surface models capture the full anatomical topology of affected regions and allow for the precise spatial localization of scars relative to joints, flexion creases, and movement-critical zones. This enables segmentation of the body surface into anatomically and functionally relevant units and supports longitudinal monitoring of scar morphology and surface reduction within specific functional regions. Future studies will therefore extend the current area-based methodology toward CFU-based metrics and correlate 3D-derived topographic parameters with objective functional outcomes [19,20].

As this was a pilot observational study, patients were not randomized, which may limit the generalizability of the findings. The sample size was small and heterogeneous, which limits statistical inference. Scanning intervals were not standardized but determined clinically, resulting in variable follow-up periods. Future studies should include larger cohorts, standardized scanning intervals, automated segmentation techniques, and a more detailed evaluation of factors influencing healing (comorbidities, perfusion, infection, treatment modality).

Although patients were consistently positioned in the same posture during all scanning sessions, complete reproducibility of cutaneokinematic conditions could not be fully guaranteed. Subtle physiological changes in skin tension or limb load, even under identical positioning, may have introduced minor variability in area measurements. This inherent variability is acknowledged as a limitation of the pilot study.

By capturing the complex dynamics of wound-area reduction and epithelialization, it provides clinically meaningful metrics that can enhance documentation, guide therapeutic decisions, and support personalized treatment strategies. These findings support the further implementation of 3D surface analysis in clinical burn care and justify larger prospective studies.

## 5. Conclusions

This pilot study demonstrated that 3D surface scanning provides a robust, non-invasive, and accurate method for the quantitative assessment of burn wound healing, with a primary focus on objective measurement of the wound surface area and its longitudinal changes. The results clearly demonstrated that these metrics can be obtained consistently even in a small and heterogeneous patient cohort, indicating that 3D scanning provides substantial added value beyond traditional visual inspection or 2D estimation.

Importantly, this study fulfilled its purpose as a methodological validation. Building on these findings, future research will focus on leveraging 3D scanning not only for area-based quantification, but also for detailed monitoring of wound topography and scar evolution. In particular, the next phase will involve evaluating the therapeutic effect of custom-made pressure orthoses by comparing the 3D healing trajectories between treated and control groups. Such an investigation will require a larger, more balanced sample and standardized follow-up intervals to reliably assess treatment efficacy using high-resolution 3D surface metrics.

Overall, this pilot work establishes a strong foundation for integrating 3D scanning into burn care and sets the stage for controlled prospective studies aimed at improving personalized treatment strategies.

## Figures and Tables

**Table 1:** Demographic and clinical data.

Patient (*n* = 18)	Gender	Age	Height	Weight	BSA (m^2^)	TBSA 3D Scan (%)
P 01	F	64	173	67	1.79	2
P 02	F	27	180	70	1.88	3
P 03	M	52	186	90	2.14	3
P 04	M	54	183	100	2.22	3
P 05	M	50	182	90	2.15	6
P 06	M	19	174	83	1.97	4
P 07	M	28	178	108	2.49	2
P 08	M	63	180	114	2.32	1
P 09	M	53	190	100	2.28	30
P 10	F	79	165	70	1.77	6
P 11	M	37	180	110	2.28	6
P 12	M	58	168	90	1.99	9
P 13	F	60	165	106	2.11	8
P 14	M	65	180	80	1.99	9
P 15	M	42	190	110	2.37	1
P 16	M	77	180	80	1.99	1
P 17	M	20	180	90	2.98	14
P 18	M	21	175	90	2.05	6

**Table 2:** Wound-level 3D area metrics.

Patient	Gender	Age	Mechanism of Burn	Burn Degree *	Location **	Period (Days)	Wound Area A_t_ (cm^2^)	ΔA (cm^2^)	Percent Reduction (%)	ΔTBSA (%)	Surgery
P01	F	64	Scald	II and III	RLL	1	329.19	230.77	70.10	1.388	1
						10	191.12				
						15	98.42				
					LLL	1	21.07	18.86	89.51		
						10	2.59				
						15	2.21				
P02	F	27	Fire	IIa	RUL	1	320.17	208.35	65.07	1.816	
						5	111.82				
					LUL	1	172.49	134.21	77.81		
						5	38.28				
P03	M	52	Fire	III	LLL	1	160.25	74.72	46.63	1.632	
						3	164.05				
						8	111.25				
						11	85.53				
					RUL	1	91.24	79.86	87.53		
						3	60.34				
						8	30.79				
						11	11.38				
					LUL	1	373.12	196.18	52.58		
						3	324.53				
						8	182.50				
						11	176.94				
P04	M	54	Scald	IIa	Torso	1	173.13	25.28	14.60	0.721	
						3	147.85				
					RUL	1	116.73	22.65	19.40		
						3	94.08				
					LUL	1	408.24	110.45	27.6		
						3	297.79				
					LLL	1	7.27	1.81	24.90		
						3	5.46				
P05	M	50	Scald	IIa	LUL	1	149.07	55.52	37.24	1.145	
						3	93.55				
					RUL	1	163.94	112.29	68.49		
						3	51.65				
					LUL	1	920.52	74.47	8.9	3.119	
P06	M	19	Explosion	IIb		3	322.40				
						11	126.43				
					RUL	1	721.13	617.12	85.58		
						3	260.27				
						11	104.01				
P07	M	28	Explosion	IIa	LUL	1	191.17	57.11	29.66	0.536	
						5	145.88				
						11	116.70				
					RUL	1	188.22	46.03	24.46		
						5	142.61				
						11	142.19				
P08	M	63	Explosion	IIb	LUL	1	192.58	57.11	29.66	0.246	
						5	135.47				
P09	M	53	Fire	I and IIb	Torso	1	1596.06	793.21	49.70	12.941	
						11	802.85				
					RUL	1	2137.98	842.04	39.39		
						11	1295.94				
					LUL	1	629.73	169.08	26.84		
						11	460.65				
					RLL	1	2058.89	934.03	45.38		
						11	1124.86				
					LLL	1	323.56	215.82	66.72		
						11	107.74				
P10	F	79	Scald	I–III	Torso	1	583.47	360.02	61.68	3.732	
						12	290.63				1
						19	223.45				
					RUL	1	329.03	167.52	50.90		
						12	143.90				
						19	161.51				
					RLL	1	93.56	77.38	82.70		
						12	33.10				
						19	16.18				
					LUL	1	86.39	56.09	64.93		
						12	21.66				
						19	30.30				
P11	M	37	Explosion	IIb and III	Torso	1	538.18	28.25	5.25	2.357	
						8	509.93				
					RUL	1	833.13	510.48	61.27		
						8	322.65				
P12	M	58	Scald	IIb	RLL	1	1502.51	1099.91	73.20	7.055	1
						3	1175.52				
						8	941.05				
						16	507.83				
						23	402.60				
					LLL	1	356.35	308.58	86.59		
						3	156.60				
						8	119.09				
						16	49.81				
						23	47.77				
P13	F	60	Scald	IIb	Torso	1	568.36	524.57	92.30	4.464	
						4	439.99				
						9	80.83				
						11	50.93				
						37	43.79				
					LUL	1	903.87	418.52	46.30		
						4	847.21				
						9	722.26				
						11	530.28				
						37	485.35				
P14	M	65	Scald	IIa	RUL	1	652.70	411.29	63.01	6.397	1
						3	473.96				
						7	286.85				
						11	241.41				
					LUL	1	1186.65	865.86	72.97		
						3	685.30				
						7	436.58				
						11	320.79				
P15	M	42	Scald	IIb	LLL	1	30.84	17.45	56.58	0.073	
						5	13.39				
P16	M	77	Fire	III	RLL	1	127.05	23.60		0.118	1
						7	125.18		18.58		
						22	103.45				
P17	M	20	Explosion	I and IIa	Torso	1	1153.70	802.07	69.52	9.196	
						7	391.96				
						10	351.63				
					RUL	1	685.07	273.16	39.87		
						7	426.92				
						10	411.91				
					RLL	1	988.16	854.92	86.52		
						7	789.28				
						10	133.24				
P18	M	21	Explosion	I and IIa	RLL	1	88.78	33.82	38.09	2.729	
						7	70.03				
						10	54.96				
					RUL	1	295.18	202.46	68.59		
						7	250.69				
						10	92.72				
					LUL	1	947.80	324.93	34.28		
						7	869.02				
						10	622.87				

* Burn depth classification: I = superficial, II = partial-thickness (IIa = superficial dermal; IIb = deep dermal), III = full-thickness. ** RUL = right upper limb, LUL = left upper limb, RLL = right lower limb, LLL = left lower limb.

## Data Availability

The original contributions presented in this study are included in the article. Further inquiries can be directed to the corresponding author.

## References

[1] Chang C.W., Wang H., Lai F., Christian M., Huang S.C., Tsai H.Y. (2025). Comparison of 3D and 2D Area Measurement of Acute Burn Wounds with LiDAR Technique and Deep Learning Model. Front. Artif. Intell..

[2] Peake M., Pan K., Rotatori R.M., Powell H., Fowler L., James L., Dale E.L. (2019). Incorporation of 3D Stereophotogrammetry as a Reliable Method for Assessing Scar Volume in Standard Clinical Practice. Burns.

[3] Farrar E., Pujji O., Jeffery S. (2017). Three-Dimensional Wound Mapping Software Compared to Expert Opinion in Determining Wound Area. Burns.

[4] Ondrejová B., Michalíková M., Štefanovič B., Bednarčíková L., Živčák J. (2024). Utilization of 3D Scanning in Burn Analysis and Identification. Lékař Tech.—Clin. Technol..

[5] Bloemen M.C.T., Boekema B.K.H.L., Vlig M., van Zuijlen P.P.M., Middelkoop E. (2012). Digital Image Analysis versus Clinical Assessment of Wound Epithelialization: A Validation Study. Burns.

[6] Teo Y., Abbas A., Park E., Barbut C., Guo J., Goh D., Yeong J., Mok W.L.J., Teo P. (2023). 3D Printed Bioactive PLGA Dermal Scaffold for Burn Wound Treatment. ACS Mater. Au.

[7] Tanner J., Rochon M., Harris R., Beckhelling J., Jurkiewicz J., Mason L., Bouttell J., Bolton S., Dummer J., Wilson K. (2024). Digital wound monitoring with artificial intelligence to prioritise surgical wounds in cardiac surgery patients for priority or standard review: Protocol for a randomised feasibility trial (WISDOM). BMJ Open.

[8] Visscher D., te Slaa S., Jaspers M., van de Hulsbeek M., Borst J., Wolff J., Forouzanfar T., van Zuijlen P.V. (2018). 3D Printing of Patient-Specific Neck Splints for the Treatment of Post-Burn Neck Contractures. Burn. Trauma.

[9] Ondrejová B., Michalíková M., Štefanovič B., Bednarčíková L., Živčák J. (2023). Technological Process of Design and Production of Facial Burn Mask. Acta Tecnol.—Int. Sci. J. About Technol..

[10] Uchida D.T., Bruschi M.L. (2023). 3D Printing as a Technological Strategy for the Personalized Treatment of Wound Healing. AAPS PharmSciTech.

[11] Cretu A., Grosu-Bularda A., Bordeanu-Diaconescu E.-M., Hodea F.-V., Ratoiu V.-A., Dumitru C.-S., Andrei M.-C., Neagu T.-P., Lascar I., Hariga C.-S. (2025). Strategies for Optimizing Acute Burn Wound Therapy: A Comprehensive Review. Medicina.

[12] Haleem A., Javaid M., Vaishya R. (2019). 3D Scanning Applications in Medical Field: A Literature-Based Review. Clin. Epidemiol. Glob. Health.

[13] Serena T., Yaakov S., Yaakov R., King E., Driver V.R. (2024). Percentage Area Reduction at Week 4 as a Prognostic Indicator of Complete Healing in Patients Treated with Standard of Care: A Post Hoc Analysis. J. Wound Care.

[14] Bull R.H., Staines K.L., Collarte A.J., Bain D.S., Ivins N.M., Harding K.G. (2022). Measuring Progress to Healing: A Challenge and an Opportunity. Int. Wound J..

[15] Kantor J., Margolis D.J. (2000). Percent Change in Wound Area as a Prognostic Indicator of Healing in Venous Leg Ulcers. Wound Repair Regen..

[16] Du Bois D., Du Bois E.F. (1916). A Formula to Estimate the Approximate Surface Area if Height and Weight Be Known. Arch. Intern. Med..

[17] Brekke R.L., Almeland S.K., Hufthammer K.O., Hansson E. (2023). Agreement of Clinical Assessment of Burn Size and Burn Depth Between Referring Hospitals and Burn Centres: A Systematic Review. Burns.

[18] Rashaan Z.M., Euser A.M., van Zuijlen P.P.M., Breederveld R.S. (2018). Three-Dimensional Imaging Is a Novel and Reliable Technique to Measure Total Body Surface Area. Burns.

[19] Parry I.S., Bell J.F., Schneider J.C., Bidwell J.T., Catz S.L., Tancredi D.J. (2025). Cutaneous functional units (CFUs) versus total body surface area burned (TBSA) for predicting range of motion outcomes: A comparison of predictive models. Burn. J. Int. Soc. Burn. Inj..

[20] Parry I.S., Bell J.F., Schneider J.C., Bidwell J.T., Catz S.L., Tancredi D.J. (2025). Using Cutaneous Functional Units (CFUs) to Understand Burn Characteristics Associated With Range of Motion at Hospital Discharge in Adult Burn Survivors. J. Burn. Care Res. Off. Publ. Am. Burn. Assoc..

